# Comparative Study of Different Diets-Induced NAFLD Models of Zebrafish

**DOI:** 10.3389/fendo.2018.00366

**Published:** 2018-07-05

**Authors:** Bo Chen, Yang-Min Zheng, Jing-Pu Zhang

**Affiliations:** Key Laboratory of Biotechnology of Antibiotics, The National Health Commission, Beijing Key Laboratory of Antimicrobial Agents, Institute of Medicinal Biotechnology, Peking Union Medical College, Chinese Academy of Medical Sciences, Beijing, China

**Keywords:** zebrafish, NAFLD, autophagy, RNA-seq analysis, overfeeding

## Abstract

Dietary composition has important impact on nonalcoholic fatty liver disease (NAFLD). The purpose of this study was to explore the relationship between NAFLD and major dietary components using zebrafish larvae fed different diets. Zebrafish larvae fed with high cholesterol (HC), high fructose (HF) and extra feeding (EF) diets for 10 days displayed varying degrees steatosis. The incidence and degree of steatosis were the most severe in the EF group. A HC diet severely promoted lipid deposits in the caudal vein. The triglyceride and glucose contents of zebrafish significantly increased in the HF and EF groups compared with the control group. Moreover, the mRNA expression of oxidative stress gene gpx1a, endoplasmic reticulum stress genes ddit3 and grp78, inflammatory genes tnfa, glucose metabolism genes irs2, glut1 and glut2, and lipid metabolism genes cidec, chrebp, ppara and cpt1a were significantly increased in the HF group. The HC diet was associated with upregulation of grp78, and downregulation of irs2, glut1 and glut2. The mRNA expression of lipogenesis and glucose metabolism associated genes were decreased in the EF group. In addition, the autophagy associated genes atg3, atg5, atg7, and atg12, and protein expression of ATG3 and LC3BII were reduced and P62 were elevated in the HC group. We also performed comparative transcriptome analysis of the four groups. A total of 2,492 differentially expressed genes were identified, and 24 statistically significant pathways were enriched in the diet treatment groups. This study extends our understanding of the relationships between diet ingredients and host factors that contribute to the pathogenesis of NAFLD, which may provide new ideas for the treatment of NAFLD.

## Introduction

Non-alcoholic fatty liver disease (NAFLD), characterized by the accumulation of excess fat in liver cells, has been increasing in prevalence in recent years (1). NAFLD is closely associated with several adverse health consequences including obesity, insulin resistance, type 2 diabetes and cardiovascular disease (2). Indeed, NAFLD is a broad classification, which ranges from steatosis alone to non-alcoholic steatohepatitis (NASH) (3). NASH may undergo progression from nonalcoholic steatohepatitis to fibrosis and may eventually lead to the development of hepatocellular carcinoma (4). Treatment approaches are limited because of the unclear pathogenesis of NAFLD and lack of therapeutic agents. Furthermore, the prevalence of NAFLD is increasing in children and adolescents who develop NAFLD mostly because of overeating. This makes research on the pathological mechanism of NAFLD more pressing, and suitable NAFLD models have induced an urgent need to study this disease in more depth (5, 6).

Dietary composition has significant impact on the development of NAFLD (7). It is well known that a high fat diet or diet rich in sugar can induce hepatic steatosis, obesity and insulin resistance (8, 9). Studies have also shown that the ingestion of a high cholesterol diet contributes to the rise of obesity and the prevalence of NAFLD, which are the major risk factors for atherosclerosis and cardiovascular disease (2, 9, 10). Today, fructose is becoming the most common consumption of sugar, and dietary fructose promotes hepatic de novo lipogenesis and subsequent hepatic steatosis (11, 12). People currently have a lack of indepth understanding of the impact of diet composition on health. Thus, it is important to clarify the differential contribution of diet to the development of NAFLD.

Traditionally, rodent models are used widely to investigate mechanisms underlying NAFLD and test candidate agents. However, the financial burden of using rodent models needs to be considered and the use of rodents does not suit large sample size screening. Recent studies have shown that the process of lipid metabolism in zebrafish is similar to that in humans (13). Interest in the application of zebrafish for *in vivo* models is increasing across various fields such as genetics, developmental biology, toxicology, and preclinical medicine experiments. The advantages of the use of zebrafish are the low financial cost, fast maturing and easy genetic modification. Moreover, the optical transparency of zebrafish larvae enables the whole body real-time monitoring (14). Recently, the potential of zebrafish in lipid metabolism research has been reported as very positive (13, 15–18). To date, several zebrafish models of NAFLD are well established including transgenic models, chemical induced models and diet induced models (19–21). Sapp et al showed that zebrafish larvae treated with fructose developed hepatic steatosis by activating ER stress and oxidative stress (20). Correspondingly, zebrafish larvae fed a high fat or high cholesterol diet exhibited lipid accumulation in the liver (21). Excessive consumption of calories by overfeeding contributed to obesity and hepatic steatosis in zebrafish larvae (22, 23). However, there is lack of a comprehensive analysis to indicate the impact of diet compositions on the development of NAFLD in zebrafish larvae.

In this study, we established three models by feeding zebrafish a high cholesterol diet, a high fructose diet, and a high calorie diet, and analyzed their differential impacts. In addition, it has been confirmed that autophagy regulates lipid metabolism by eliminating triglycerides, and plays a key role in the development of NAFLD (24, 25). However, little is known about autophagy in diet-induced NAFLD in zebrafish. We further assessed the autophagy activities in these models. We aim to provide an insight into the effects of major dietary components in zebrafish, which will be helpful in advancing our understanding of the pathogenesis of NAFLD.

## Materials and methods

### Zebrafish care and feeding

Zebrafish (Danio. rerio) of wild-type AB strain and transgenic GFP-LC3 (26) were raised under standard laboratory conditions with 14-h light/10-h dark cycle at a temperature of 28.5 ± 1°C (27). Embryos and larvae were maintained in embryo medium to 5 dpf. The fish were randomly divided into four groups to be fed with different diets: 30 mg/d AP100 (Larval-AP100, Zeigler Bros, Inc., USA) as the control group, 30 mg/d AP100 plus 4% (w/w) cholesterol as the HC group, 30 mg/d AP100 plus 0.25%(w/v) fructose as the HF group, 180 mg/d AP100 as the EF group. Two-hundred fish were included in each group and began feeding at 5 dpf for 10 days. All zebrafish larvae were fasted overnight before sacrifice at the end of the study. For the aim of studying cholesterol transport in larvae, all the foods were supplemented with 10 μg/g of a fluorescent cholesteryl ester analog (cholesteryl BODIPY 542/563-C11 from Invitrogen). The body length of the zebrafish from the front end of the mouth to the tail end was measured, and body mass was weighed using an electronic balance scale (BS2202S, Sartorius, Argentina, Germany). This research was reviewed and approved by the Laboratory Animal Management and Animal Welfare Committee in Institute of Medicinal Biotechnology of Chinese Academy of Medical Sciences and the most effort was made to minimize the animals' suffering.

### Whole-mount oil red O staining

Zebrafish larvae were fixed in 4% paraformaldehyde (PFA) overnight at 4°C and washed twice with phosphate-buffered saline (PBS). ORO staining was performed as described previously (20). Lipid droplets in liver tissue were observed and imaged on a bright-field dissecting microscope (Olympus szx10, Tokyo, Japan). Larvae were defined positive for steatosis according to the previous report (20).

### Histologic analysis

Larvae were fixed with 4% PFA overnight, coated in paraffin and cut into 4 μm slices with paraffin microtome. The sections were stained with hematoxylin and eosin (H&E). For ORO staining of cryosections, larvae were embedded in OCT (Sakura Ltd., Tokyo, Japan), and stored at −80°C until sectioning. Serial sections (8 μm) were cut. Each section was stained with 0.5% ORO. Slides were observed using an Olympus BX53 microscope (Olympus, Tokyo, Japan), and the positive area of the ORO was calculated using Image J software.

### Biological analysis

Levels of total cholesterol (TC) and triglycerides (TGs) of the dissected of zebrafish larvae livers were determined using the Total Cholesterol Reagent Kit and Triglyceride Reagent Kit (Applygen Technologies Inc., Beijing, China) according to the manufacturer's specification.

### Quantitative RT-PCR

Total RNA was isolated from dissected livers of zebrafish larvae by TRIzol reagent (No. 15596026, Thermo Fisher, USA) and then converted into complementary DNA (cDNA) by reverse transcriptase (No.M1701, Promega, USA). The cDNAs were then introduced as the templates for quantitative real-time polymerase chain reaction (qPCR). The reaction of qPCR was performed by the real-time quantification system (ROCHE LightCycler 96, Switzerland). The relative amount of messenger RNAs (mRNAs) was calculated with β-actin mRNA as the invariant control. The relative transcript expression level was determined using the control sample as a calibrator and the ΔΔCT method. The following specific primers were used for amplification in this study (Table S2).

### Western blotting

Dissected livers of zebrafish larvae (20–30 in each group) were washed with ice-cold PBS and lysed directly by lysis buffer (Applygen Technologies Inc., Beijing, China). The protein samples were blotted and incubated with ATG3 (SC-70139, Santa Cruz Biotechnology, Texas, USA), LC3B (PM036, MBL, Japan), p62 (PM045, MBL, Japan), β-actin (A5441, Sigma-Aldrich) and secondary HRP-conjugated goat anti-mouse or goat anti-rabbit secondary antibodies (ZSGB-BIO, Beijing, China). The protein bands were detected with SuperSignal West Pico chemiluminescent substrate (34080, Thermo Fisher, Waltham, MA).

### RNA-seq, GO, and KEGG analysis

Total RNAs were extracted from the liver tissues of zebrafish larvae. A total amount of 1.5 μg RNA per sample was used as input material for the RNA sample preparations. The methods were particularly described. Briefly, sequencing libraries were generated by using NEBNext® UltraTM RNA Library Prep Kit for Illumina® (NEB, USA). The products were purified (AMPure XP system) and library quality was analyzed using the Agilent Bioanalyzer 2100 system. For high-throughput sequencing, the library preparations were applied to Illumina Hiseq 4000 platform for 150 bp paired-end sequencing by Compass Biotechnology Inc. (Beijing, China). Following analysis for raw data (Fastq fles) was done by Compass Biotechnology Inc. Then based on the KEGG and GO database (28), biomedical pathways were classified. For enrichment statistics, the whole genes were identified in the RNA-seq analysis, which was set as background. The gene expression and pathway differences were considered statistically significant with a *p*-value of 0.05.

### Statistical analysis

Data are expressed as means ± SEM. For multiple group comparisons, one-way ANOVA with a Tukey's *post-hoc* test was performed. For all experiments, *P* < 0.05 was considered statistically significant.

## Results

### Assessment of the body length and body weight in the HC, HF, and EF diets-fed zebrafish larvae

**Figure 1:**
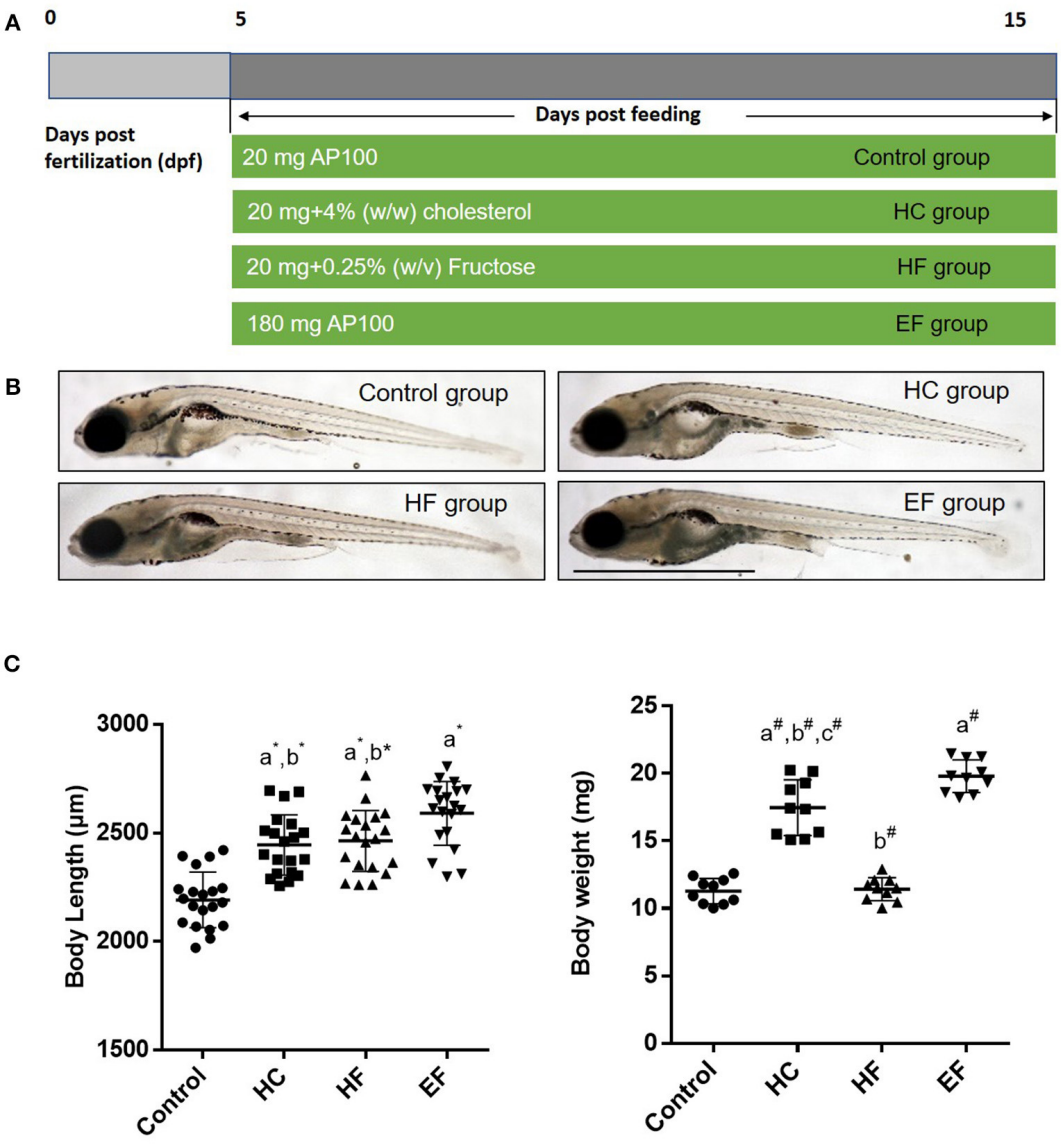
Effects of HC, HF, and EF diets feeding on the growth of zebrafish larvae after 10 days feeding. **(A)** Description of the diets feeding protocol. **(B)** General observation of zebrafish in three groups. **(C)** Body length was measured at 15 dpf (*n* = 20). Ten fish for each sample were weighted from 100 fish per group at 15 dpf (*n* = 10). a, compared with control; b, compared with EF; c, compared with HF; *P < 0.05, ^#^*P* < 0.01. Bar = 1 mm.

To develop a model of liver steatosis, 5 dpf zebrafish larvae were treated with control diet, HC diet, HF diet and EF diet for 10 days (Figure 1A). Our preliminary experiments showed that the fructose concentration at higher than 0.25% (w/v) could significantly increase the death rate of zebrafish larvae after 10 days treatment. So, we stabilized the concentration of fructose at 0.25 (w/v). Compared with the control group, the larvae body length in the HC, HF, and EF groups were significantly larger (*P* < 0.01). Furthermore, the larvae body length was longer in the EF group compared to the HC and HF groups (*P* < 0.01, *P* < 0.05, respectively), but there was no significant difference between the HC and HF groups (Figures 1B,C). Since one single larva was too light to be measured, we defined 10 larvae as one sample to measure body weight. The body weight in the HC and EF groups was significantly heavier than in the control and HF groups (*P* < 0.01). The HC group weighed significantly less than the EF group (*P* < 0.01), and no obvious differences were observed between the control and HC groups (Figure 1C).

### HC, HF and EF diets induced liver fat accumulation in zebrafish larvae

**Figure 2:**
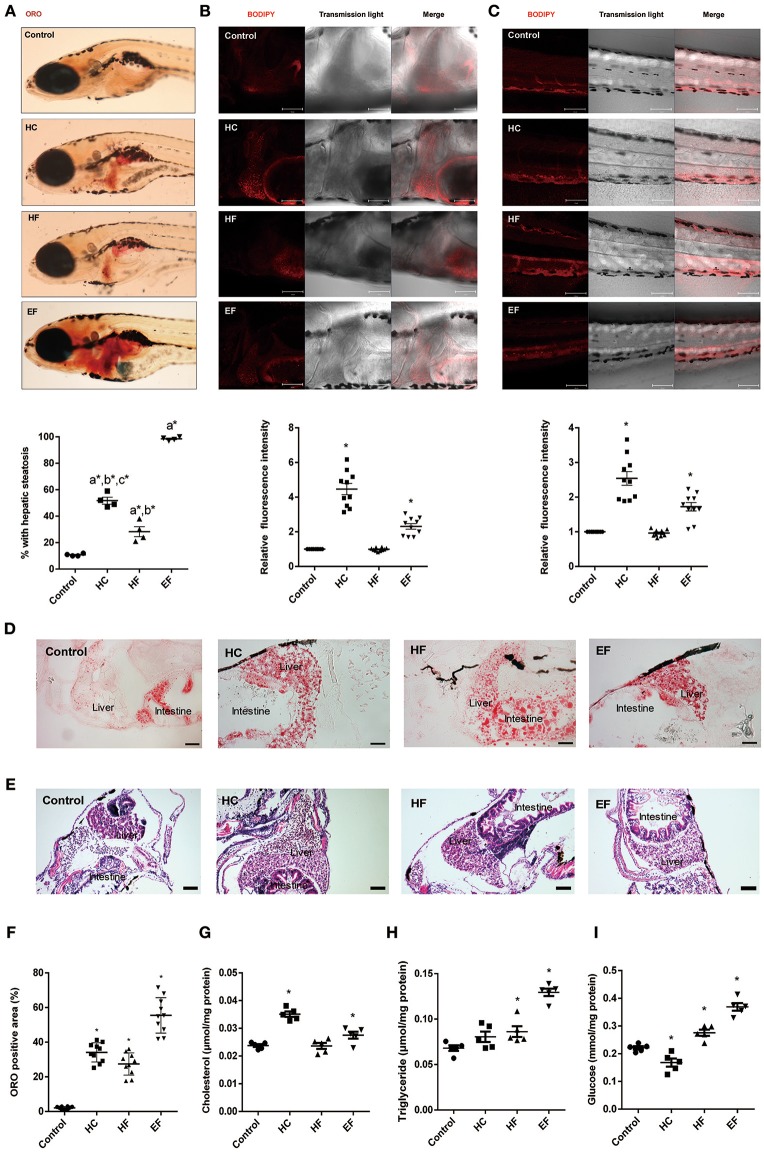
Assessment of lipid accumulation in zebrafish larvae. **(A)** The hepatic steatosis was observed by whole body ORO staining (**A**, below). The percent of larvae with hepatic steatosis was calculated from at least 100 fish each group. Data were representative of four independent experiments. **(B)** Showed fluorescent lipid accumulation deposits in the liver. Relative fluorescence intensities were quantified in the areas (*n* = 10). **(C)** Showed fluorescent lipid accumulation deposits in the blood vessel wall. Relative fluorescence intensities of the areas were quantified (*n* = 10). **(D)** Numerous lipid droplets in the hepatocytes were observed by ORO staining of frozen liver sections in HC, HF, and EF groups. The statistical results of positive area of ORO staining was showed in figure **(F)** (*n* = 10). **(E)** The lipid vacuoles were observed by H&E staining in HC, HF, and EF groups. Changes of cholesterol **(G)**, triglyceride **(H)**, and glucose **(I)** in livers of zebrafish larvae fed with control, HC, HF, and EF diets. Each sample were collected from 20 to 30 fish (*n* = 5). a, compared with control; b, compared with EF; c, compared with HF; *P < 0.05 vs. Control. Bar = 100 μm.

Oil Red O (ORO) stain was employed to detect the lipid accumulation in zebrafish larvae. We found that about 10% of zebrafish developed steatosis even in the control group. HC, HF, and EF diets all induced liver steatosis at a significant level (Figure 2A). The ORO staining of frozen larvae sections treated with HC, HF, and EF diets showed substantial fat accumulation in the form of small and large lipid droplets in hepatocytes (Figures 2D,F). The livers in the HC and HF groups displayed mild lipid accumulation and foci of microvesicular steatosis. Without apparent necrosis, zebrafish given an EF diet developed obvious liver lipid accumulation and severe macro-vascular steatosis (Figures 2D,E). Interestingly, using a fluorescent cholesteryl ester analog, we observed that larvae fed with a HC diet developed remarkable lipid deposits in the liver and caudal vasculature, and its fluorescence intensity was much higher than in the HF and EF groups (Figures 2B,C). Furthermore, we examined the level of triglyceride (TG), total cholesterol (TC) and glucose (GL) in the liver of zebrafish larvae. The levels of TC, TG and GL were significantly higher in the EF group compared to the control group. Treatment with a HC diet was significantly associated with higher TC levels, but reduced GL levels (Figures 2G–I). Larvae fed with a HF diet obviously had elevated TG and GL levels, but TC levels were not elevated (Figures 2G–I). These results demonstrate that HC, HF and EF diets exert different degrees of action in liver steatosis and vessel lipid deposits.

### Transcription changes of ER stress, oxidative stress and inflammatory genes in HC, HF, and EF diets fed zebrafish larvae

**Figure 3:**
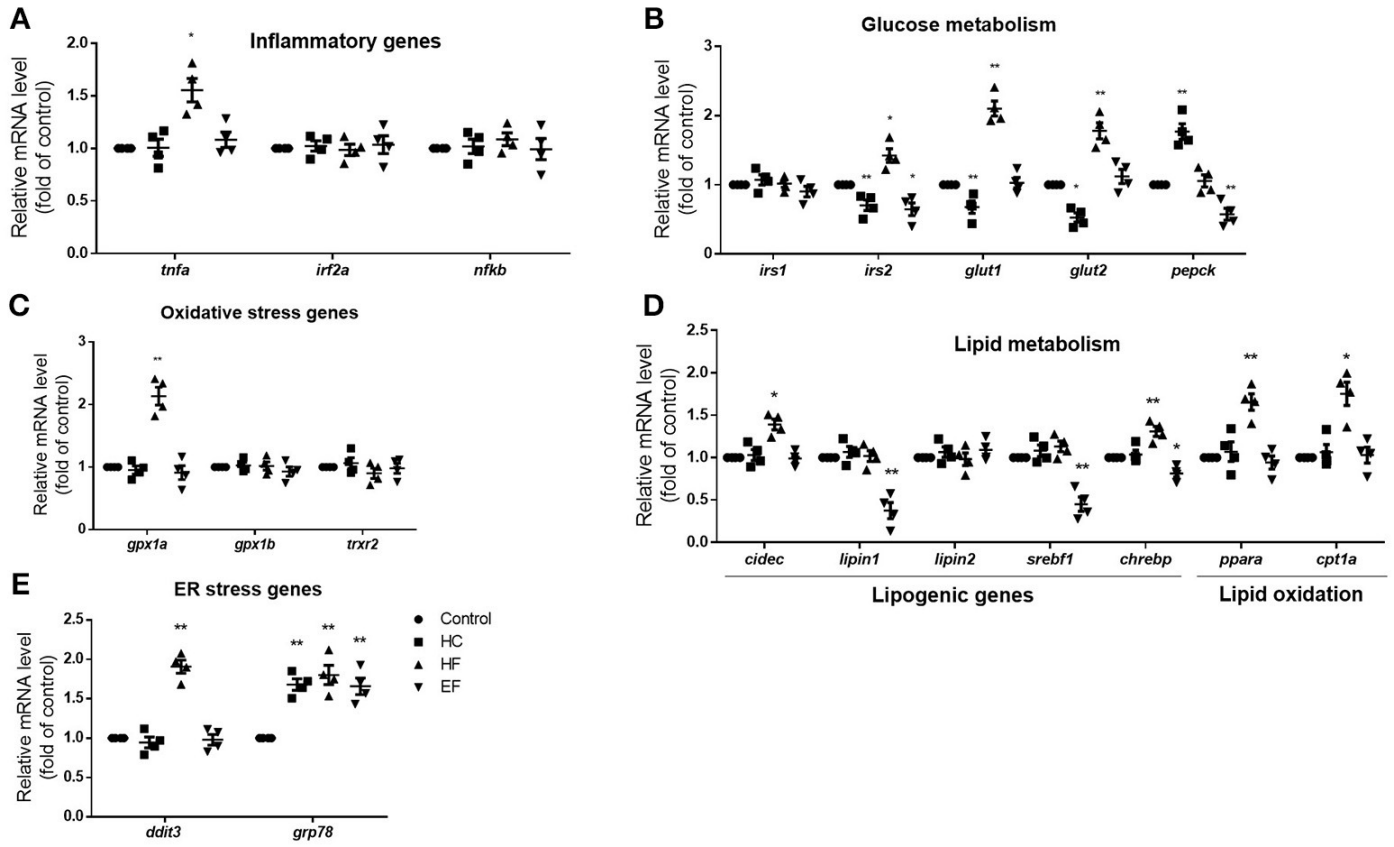
Changes of NAFLD associated genes in HC, HF, and EF diets fed zebrafish larvae. The genes expression involved in **(A)** inflammation **(B)** glucose metabolism, **(C)** oxidative stress, **(D)** lipid metabolism, and **(E)** ER stress in control, HC, HF, and EF groups were measured by qPCR (*n* = 4). *P < 0.05, **P < 0.01 vs. control.

The pathogenic factors such as inflammation, oxidative stress and ER stress are thought to induce liver damage. To further analyze the characteristics of the three models, qPCR was applied to detect the mRNA expression level of genes related to inflammation (tnfa, irf2a, and nfkb), oxidative stress (gpx1a, gpx1b, trxr2) and ER stress (ddit3, grp78). The transcription level of inflammatory gene tnfa and oxidative gene gpx1a were elevated in the HF diet group, but there were no significant differences between the HC and EF group. However, the ER stress gene grp78 in the three diets-treated groups was higher compared to the control group. The mRNA expression of ddit3 was also significantly elevated in the HF group (Figures 3A–C). The above results, along with the morphologic and biochemical results, indicated that fructose may be involved in the activation of inflammatory pathways, oxidative stress, and ER stress. Cholesterol and excessive calories may contribute to ER stress but not inflammatory pathways or oxidative stress.

### Transcription changes of lipid metabolism and glucose metabolism associated genes in HC, HF, and EF diets fed zebrafish larvae

The liver is a key organ in human lipogenesis and gluconeogenesis. Dysregulation of liver glycolipid metabolism is an important cause of NAFLD. We observed the gene expression related to lipid and glucose metabolism. Compared with the control group, the expression level of lipogenic genes, lipin1 and srebf1 were significantly lower in the EF-treated group. The results implied excessive calories uptake which may suppress endogenous lipogenesis. HF diet treated fish showed an increase in expression of lipogenesis genes cidec and chrebp, the lipid oxidation genes ppara and cpt1a, which suggested that fructose may promote lipogenesis and fatty acid β-oxidation. An interesting thing is, HC-treated larvae showed down-regulation of irs2a, glut1 and glut2. EF-treated larvae also showed down-regulation of irs2a. However, HF-treated larvae exhibited up-regulation of glut1 and glut2 compared to the control group (Figures 3D,E). These results demonstrated that high cholesterol levels may impact glucose transport and insulin sensitivity. It is not surprising that fructose influences the gene expression of glucose metabolism.

### Effects of different dietary components on autophagy in zebrafish larvae

**Figure 4:**
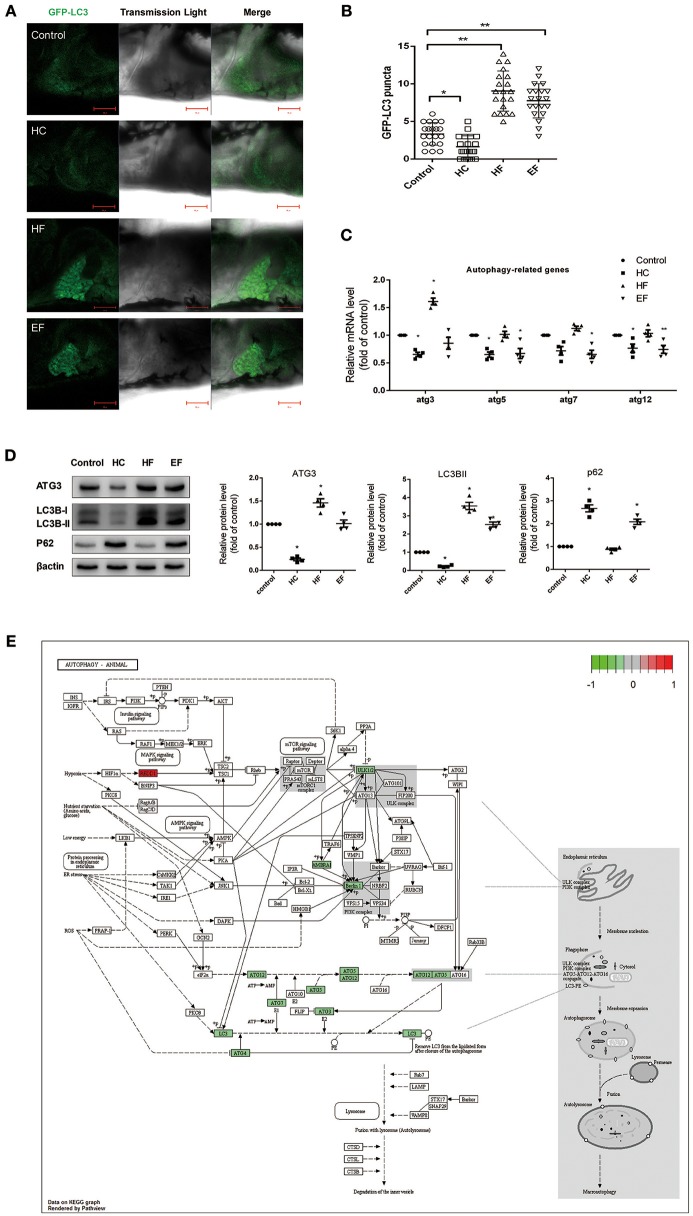
Effects of HC, HF, and EF diets treatment on autophagy. **(A)** LC3 puncta formation in liver of *GFP-LC3* transgenic zebrafish larvae fed with control, HC, HF, and EF diets. Bar = 100 μm. **(B)** Quantification of LC3 puncta abundance in **(A)** with software Image J. **(C)** The genes expression involved in autophagy (*n* = 4). **(D)** The protein expression of LC3BII, ATG3, and p62 were detected by western blot in liver of zebrafish larvae fed with control, HC, HF, and EF diets (*n* = 4) *P < 0.05, **P < 0.01 vs. control. **(E)** Autophagy pathway in HC group by KEGG. Genes with red-filled are up-regulated, while genes with green-filled are down-regulated.

It has been reported that autophagy participates in regulating intracellular lipid metabolism and the development of NAFLD. However, little is known about the interactions between autophagy and pathology of NAFLD induced by over consumption. We performed qPCR to detect mRNA levels of autophagy related genes in zebrafish treated with HC, HF, and EF diets. Compared with the control group, zebrafish larvae treated with a HC-diet exhibited significantly decreased expression of atg3, atg5, and atg12 as well as a mildly decreased expression of atg7. Larvae treated with a HF-diet showed a significant up-regulation of atg3. Similar to the HC group, fish treated with an EF diet demonstrated decreased expression of atg3, atg5, atg7, and atg12 (Figure 4C). For real-time monitoring of autophagy, GFP-LC3 zebrafish were employed under the same condition. Results showed that the number of LC3-puncta in HF groups was larger than in other groups. In HC group, the number of LC3-puncta was less than in the control group. The number of LC3-puncta in the EF group was greater than in the control group (Figures 4A,B). On the autophagy related protein level, compared to the control group, HC diets treated zebrafish throughout showed down-regulation of ATG3 and LC3BII, however P62 was elevated, indicating a blocked autophagic flux. In contrast, ATG3 and LC3BII were elevated, but P62 declined in the HF group, indicating a normal autophagy flux. Autophagy activity in the EF group was between that of the HC and HF groups, but lower than the control group (Figure 4D).

### Analysis of RNA-seq, GO analysis and significant pathway enrichment by KEGG in treated zebrafish larvae

**Figure 5:**
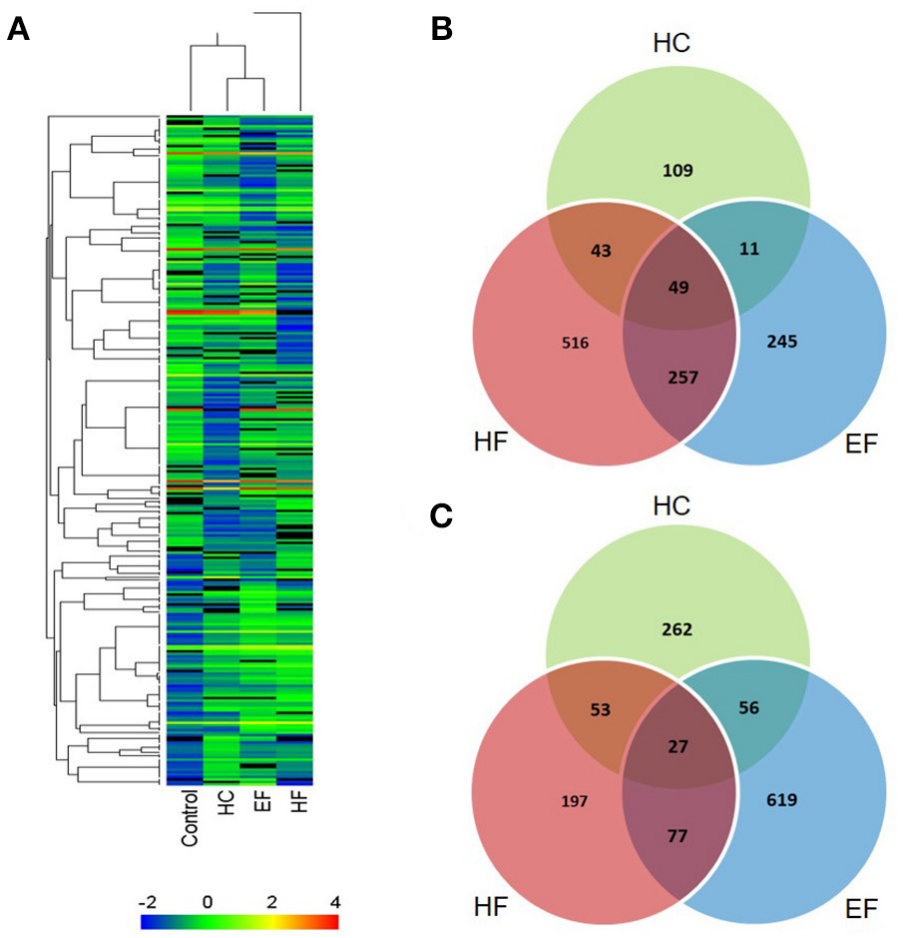
Summary of DEG analysis. **(A)** Cluster analysis of genes and samples from DEGs. **(B)** Comparison of up-regulated DEGs between different groups. **(C)** Comparison of down-regulated DEGs between different groups.

The results above showed that all three diets can cause hepatic steatosis, and the familiar NAFLD associated genes were detected. For a more comprehensive analysis, RNA-seq analysis was applied to discover new biological pathways. Compared to the control group, a total of 2,492 differentially expressed genes (DEGs) were identified in the diet treatment groups. There were 610, 1,219, and 1,341 DEGs in HC, HF, and EF groups, respectively. Among these, 212, 865 and 562 genes were upregulated, as well as 398, 354, and 779 genes were down-regulated in the HC, HF, and EF groups, respectively (Figures 5B,C). Cluster analysis of DEGs showed that the HC and EF groups displayed similar expression patterns compared to the control group, and the HF group exhibited different expression patterns compared to the other groups (Figure 5A).

When we analyzed the data based on the Kyoto Encyclopedia of Genes and Genomes (KEGG) database to classify functional annotations of DEGs in zebrafish larvae exposed to the different diets, the DEGs of the three groups were enriched mainly via 24 KEGG pathways (Table S1).

**Figure 6:**
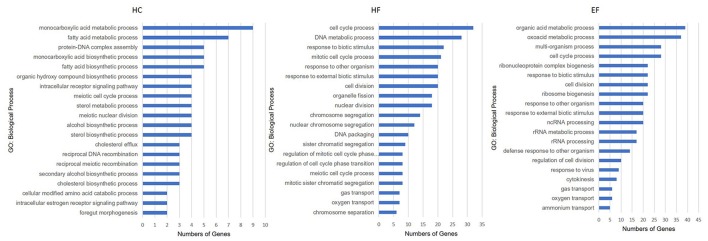
Gene ontology analysis of zebrafish larvae gene collection. Genes were categorized according to specific biological processes. Significantly enriched GO terms for DEGs of HC, HF, and EF diets treatment groups (Top 20, *P* < 0.05).

The HC group exhibited six significant pathways, with steroid biosynthesis the most significant pathway, and linoleic acid metabolism was also involved. The HF group had 10 significant pathways, with steroid biosynthesis and cell cycle the top two pathways. The EF group had 17 significant pathways, with ribosome biogenesis in eukaryotes and pyrimidine metabolism as the top two pathways, and the fatty acid metabolism related pathways, alpha-linolenic acid metabolism, PPAR signaling pathway were significantly changed in the HF and EF groups (Table S1). Although the autophagy pathway was not identified as a significant pathway in the HC groups, we found that several genes (atg3, atg4, atg5, atg7, atg12, beclin1, lc3b, ambra1, and ulk1/2) changed and were involved in the autophagosome initiation, so it seems that a HC diet had a systematic effect on autophagy (Figure 4E). GO and biological process enrichment analyses showed mostly similar results. Lipid metabolism, cell cycle process and organic acid metabolic process were most enriched in the HC, HF, and EF groups, respectively (Figure 6).

## Discussion

Here, we used three different diets to model development of NAFLD in zebrafish larvae. Although they all caused liver steatosis, the mechanisms were not exactly same. We defined a 30 mg/day/100 fish as a basic diet, based on the standard, and the mortality rate and the percentage of liver steatosis of less than 10%. On the basis of this basic diet, 4% cholesterol or 0.25% fructose were added. We found that 180 mg/day/100 fish was an excessive dose which can simulate a calorie-laden diet. Therefore, using this kind of grouping is beneficial to identify the effects of nutrient content on the development of NAFLD.

In this study, the incidence and degree of liver steatosis were the most severe in the EF group, suggesting that a high-calorie diet promotes hepatic lipid accumulation. Moreover, EF diet-fed larvae and HC diet-fed larvae also caused lipid deposits in the caudal vein. In particular, the degree of vasolipid accumulation in the HC group was much higher than the other groups, revealing that the participation of cholesterol in low-calorie diets is sufficient to induce lipid accumulation in the liver and blood vessels. Compared to mammal models, the HC-diet model of zebrafish may be suitable for studying early stage atherosclerosis (29). On the other hand, the uptake of fructose did not affect the weight or cholesterol levels, implying that cholesterol metabolism may be not dominant in liver steatosis caused by fructose. Moreover, all three diets increased the body length of larvae, suggesting that nutrient substance and high-calorie diets accelerate the skeletal development of zebrafish larvae.

With regard to the gene changes in zebrafish treated with the three different diets, our results showed that the fructose diet significantly increased mRNA levels of genes involved in inflammation tnfa, oxidation stress gpx1a and ER stress ddit3 and grp78. Sapp et al reported that a 4% (w/w) fructose induced NASH model in7dpf zebrafish led to liver steatosis independent of lipid metabolism. In the present study, we included concentration of fructose of 0.25% (w/v) in order to ensure the survival rate of larvae for the long term experiment and found similar results which demonstrated that low concentration and long-term fructose ingestion could activate inflammatory, oxidative stress and ER stress pathway. These pathogenic factors can induce liver damage and drive the progression of NAFLD to NASH (3). However, HC and EF-diets both increased the ER stress gene grp78. Since the role of ER stress in NAFLD is not well understood (30), zebrafish may be an attractive model for ER stress studies.

The liver is the core organ for adipose and glycogen synthesis. We examined the gene changes involved in glucolipid metabolism in the three models and further analyze the effects of diet ingredients. HC diet-fed larvae showed significant decreases of glut1 and glut2 and significant elevation of pepck. Glut1 and glut2 are responsible for the uptake of blood glucose in the liver (31, 32). Overexpression of PEPCK in mice led to aggravation of insulin resistance (33). The above evidence implies that a high cholesterol diet may promote insulin resistance in zebrafish, whereas PEPCK knockout mice showed liver fat accumulation and steatosis (34). Since an EF-diet significantly suppressed pepck mRNA expression, this may be one of the factors driving the most serious steatosis of the three groups. Besides, lipogenic genes were significantly reduced in EF-diet fed larvae, suggesting that excessive fat inhibited the hepatic lipid anabolic pathway. Furthermore, a HF-diet obviously stimulated the genes involved in lipid oxidation and lipogenesis, suggesting that fructose may promote fatty acid metabolism and circulation in zebrafish.

Autophagy-lysosome is one of the important pathways for lipid degradation. Autophagic dysfunction increases cellular lipid storage, which is closely related to human diseases with excessive lipid accumulation such as NAFLD (25). However, little is known about autophagy in NAFLD models of zebrafish and the effects of nutrient content on autophagy. The HC-diet significantly suppressed the mRNA expression involved in the formation of autophagosomes. In particular, the protein level of LC3BII, a marker of autophagosomes (35), was also significantly reduced, implying that a cholesterol-diet may inhibit the formation of autophagic double-membrane vesicles in zebrafish larvae. The protein levels of LC3BII and P62 were both elevated in EF-diet fed larvae, and this indicated that lipid accumulation may lead to autophagic flux damage. Nevertheless, HF-diet fed larvae showed an obvious increase in ATG3 and LC3BII and reduced P62. This may due to oxidative stress-induced autophagy, which may be one of the reasons for the nearly normal result in the HF group. Conversely, the autophagic injury caused by HC and EF-diet feeding may result in lipid over accumulation and influence lipid metabolism. For real-time, simultaneous monitoring of autophagic activation, GFP-LC3 transgene zebrafish were employed to further confirm the above results.

Beside the traditional biomedical approach, we were interested in uncovering novel targets of NAFLD in the zebrafish models. An RNA-seq, and one high-throughput sequencing method was employed to investigate the transcriptome responses of zebrafish larvae to the different diets. Based on KEGG, the DEG were grouped into several pathways. We noted that, in addition to the familiar NAFLD pathway, a HF diet also affected cell cycle and p53 pathways, which may be induced by stress signals and closely related to tumorigenesis (36). The EF diet had more impact on amino acid metabolism, which was responsible for making proteins. While the HC diet majorly affected lipid and carbohydrate metabolism. These results further demonstrated that the three diets impact different biological processes. Therefore, nutritional intake should be taken into account for drug discovery studies and treatment strategies for NAFLD.

In conclusion, this study investigated the relationship between diet ingredients and host factors that contribute to the pathogenesis of NAFLD. The results suggested that all three diets were sufficient to induce hepatic steatosis and revealed the relationships between and differences among the three kinds of diets with regard to phenotype, histology, genetic expression, and biological pathway. The most surprising findings were the fatty deposits in the blood vessel and the changes in autophagy in the treated larvae. Our study may provide insight into further application of zebrafish models and new markers, as well as mechanisms to prevent and treat NAFLD.

## Ethics statement

Institutional Animal Care and Use Committee, IACUC. All experimental procedures were conducted in conformity with institutional guidelines for the care and use of laboratory animals, and protocols were approved by Institutional Animal Care and Use Committee. No endangered animal species were involved in the study.

## Author contributions

J-PZ conceived and designed the project. BC and J-PZ wrote the manuscript. BC performed the experiments of qPCR, western blot and data processing. Y-MZ did the experiments of histologic analysis and biological analysis.

### Conflict of interest statement

The authors declare that the research was conducted in the absence of any commercial or financial relationships that could be construed as a potential conflict of interest.

## Abbreviations

HC: high cholesterol
HF: high fructose
EF: extra food
KEGG: Kyoto Encyclopedia of Genes and Genomes
GO: gene ontology
ER: endoplasmic reticulum
NAFLD: nonalcoholic fatty liver disease
DEG: differentially expressed genes
GFP: green fluorescent protein
ORO: Oil red O
PBS: phosphate-buffered saline
PFA: paraformaldehyde
TC: total cholesterols
TG: triglyceride
GL: glucose
PCR: polymerase chain reaction.
