# Supplementary material for: Comparative Study of Different Diets-Induced NAFLD Models of Zebrafish
Source: Front Endocrinol (Lausanne). 2018 Jul 5;9:366. doi: 10.3389/fendo.2018.00366 (PMC6041386; doi:10.3389/fendo.2018.00366)
Supplement: Supplementary file 1 [file Table_1.DOCX]

Supplementary Material

Comparative study of different diets-induced NAFLD models of zebrafish

**Bo Chen****#, Yang-Min Zheng#, Jing-Pu Zhang***

*** Correspondence:**Corresponding Author: Jing-Pu Zhang
[zhangjingpu@imb.pumc.edu.cn](mailto:zhangjingpu@imb.pumc.edu.cn)

# Supplementary Tables

**Table. S1** Most of significant changes pathway in HC, HF and EF diet fed zebrafish by KEGG analysis.

| **KEGG pathway** | **HC** | **HF** | **EF** |
| --- | --- | --- | --- |
|  | (*p*-value) | | |
| Steroid biosynthesis | 5.98E-05 | 1.87E-07 |  |
| Arachidonic acid metabolism | 1.65E-02 | 6.59E-03 | 3.07E-03 |
| Nitrogen metabolism | 1.68E-02 |  |  |
| Primary bile acid biosynthesis | 2.04E-02 | 4.60E-03 |  |
| Linoleic acid metabolism | 2.04E-02 |  | 7.58E-03 |
| Amino sugar and nucleotide sugar metabolism | 2.21E-02 |  |  |
| Cell cycle |  | 1.14E-07 | 6.49E-03 |
| Steroid hormone biosynthesis |  | 7.19E-03 |  |
| Retinol metabolism |  | 1.00E-02 |  |
| p53 signaling pathway |  | 1.20E-02 | 7.68E-03 |
| Mucin type O-glycan biosynthesis |  | 1.31E-02 |  |
| alpha-Linolenic acid metabolism |  | 2.06E-02 | 2.98E-02 |
| PPAR signaling pathway |  | 2.68E-02 | 4.63E-03 |
| Ribosome biogenesis in eukaryotes |  |  | 3.09E-05 |
| Pyrimidine metabolism |  |  | 2.05E-04 |
| Purine metabolism |  |  | 1.31E-03 |
| Progesterone-mediated oocyte maturation |  |  | 2.52E-03 |
| Oocyte meiosis |  |  | 7.27E-03 |
| Tryptophan metabolism |  |  | 1.16E-02 |
| Arginine biosynthesis |  |  | 1.42E-02 |
| RNA polymerase |  |  | 2.37E-02 |
| Arginine and proline metabolism |  |  | 2.48E-02 |
| Selenocompound metabolism |  |  | 2.55E-02 |
| Cysteine and methionine metabolism |  |  | 4.17E-02 |

**Table.S2** Primers for qPCR of zebrafish genes used in this study

| *cidec-F* | GACTCAGCACTCCAGACC |
| --- | --- |
| *cidec-R* | TTCCAGTTCCATCCTCATCC |
| *lipin1-F* | TCACCGAGTAAGCCAGAAC |
| *lipin1-R* | TAGGACTAAGCAGCACAGG |
| *lipin2-F* | GTGATGAGGAAGGGAAGG |
| *lipin2-R* | TGGTATTGTGTTGTGATGC |
| *srebf1-F* | AGTTCTCCGACGCTCTTG |
| *srebf1-R* | TGACCACCACCACCATTG |
| *tnfa-F* | TGCTGCCGTCTGCTTCAC |
| *tnfa-R* | GCCTGGTCCTGGTCATCTC |
| *irf2a-F* | GGAGGACATTACACCAGACAG |
| *irf2a-R* | CTACTTCAACAACGGCACAC |
| *nfkb-F* | GTAAGGGCTCGTTCTCTCAG |
| *nfkb-R* | CAGGTTAGTGTCGCAGTAGG |
| *gpx1a-F* | ACCCTGTGTCCCTTATGG |
| *gpx1a-R* | TGCTGTACCTCTTGAATGG |
| *gpx1b-F* | CAACCAGTTCGGCTATCAGG |
| *gpx1b-R* | AACGGCATTGGCTCATCG |
| *trxr2-F* | AACAAGCGTGGCAAAGAG |
| *trxr2-R* | CAGTAGGGCAAAGAGAACAG |
| *ddit3-F* | GCGAAGGTGGTGTTGGTGAC |
| *ddit3-R* | GCTGGCGGCTGGAGATGG |
| *grp78-F* | CCATTACCAACGACCAGAAC |
| *grp78-R* | CTTGTCCTCATCAGCGAATC |
| *ppara-F* | AGCGTAATCCACTCTCTG |
| *ppara-R* | GCGTCTTCTGTCTTGTTG |
| *cpt1a-F* | GAACCTCACGCTAATCTACC |
| *cpt1a-R* | TGAAGGCATCTGGACTGG |
| *irs1-F* | GCGGCACAGTTCAGAGAC |
| *irs1-R* | CGGTAGAAGCAGCAGAGG |
| *irs2-F* | TTCATCCACCACCACAGTTG |
| *irs2-R* | GACTCATACTCCTCATCAGAACC |
| *pepck-F* | GCAACACACTCTTCACCAAC |
| *pepck-R* | GTCAGCGTCACTCCTTCAG |
| *glut2-F* | GCAGAAGAACCCTCACTC |
| *glut2-R* | TCTCCGCCACAATAAACC |
| *glut1-F* | CGAGAGCAGACAGATGATG |
| *glut1-R* | ATGATGGCGATGAAGATGG |
| *atg3-F* | ACCGTCACCATTGAGAATC |
| *atg3-R* | AGTTCACCTCCTCCTTCC |
| *atg5-F* | CAACTGTGGATGGGTCTG |
| *atg5-R* | GAGCGTCTGGATGAATGG |
| *atg7-F* | AGAGTCCAGTCCGATGTC |
| *atg7-R* | GAAGTAACAGCCGAGACG |
| *atg12-F* | CCAGTTCATCTCACGCTTCCTC |
| *atg12-R* | TGCCGTCACTTCCGAAACAC |
| *chrebp-F* | TGTCACTCCGTCTCCTCTC |
| *Chrebp-R* | GCACTGATGTCTTCTTGTTGG |
| *βactin-F* | CCGTGACATCAAGGAGAAG |
| *βactin-R* | ATACCGCAAGATTCCATACC |
